# Transformer-augmented dual-branch siamese tracker with confidence-aware regression and adaptive template updating

**DOI:** 10.1038/s41598-026-35692-2

**Published:** 2026-01-13

**Authors:** K. S. Sachin Sakthi, Jae Hoon Jeong, Woo Young Choi

**Affiliations:** 1 Department of Control and Instrumentation Engineering, Pukyong National University, 45 Yongso-ro, Busan, 48513 South Korea; 2 College of Computer and Software, Kunsan National University, 558 Daehak-ro, Gunsan-si, Jeollabuk-do 54150 South Korea

**Keywords:** Visual tracking, Transformer, Siamese network, Confidence aware branch, Regression distribution learning, Template update, Engineering, Mathematics and computing

## Abstract

Visual object tracking using Siamese networks has proven effective by matching a reference target with candidate regions. However, their performance is limited by static templates, insufficient context modeling, and weak multi-level feature integration, especially under occlusion, background clutter, and appearance variation. To address these limitations, we propose TSDTrack, a transformer-augmented Siamese tracker designed for quality-aware and robust tracking. Our framework employs a ResNet backbone to extract multi-scale hierarchical features, which are fused using a transformer-based module that applies global attention to enhance semantic and spatial consistency. The prediction head consists of two branches: a confidence aware branch (CAB) that assesses the confidence of classification responses, and a regression distribution learning (RDL) branch that models bounding box localization as discrete probability distributions, improving precision under uncertainty. Furthermore, we introduce a confidence-gated template update strategy that selectively refreshes the target representation based on the CAB score, enabling adaptive appearance modeling while avoiding drift. Experiments on LaSOT, GOT-10k, OTB100, and UAV123 demonstrate that TSDTrack achieves state-of-the-art performance in both accuracy and robustness, achieving 55.5% success on LaSOT, 67.5% AO on GOT-10k, 71.6% AUC on OTB100, and 66.4% success on UAV123, outperforming recent transformer-based and Siamese trackers.

## Introduction

Visual object tracking (VOT) is a core task in computer vision that involves continuously localizing a target object across a video sequence, given only its initial position. It plays a vital role in numerous real-world applications, including autonomous driving^1,2^, surveillance, human-computer interaction^3^, robotics, and aerial inspection using UAVs. In these scenarios, trackers must cope with diverse challenges such as occlusion, background clutter, rapid motion, and drastic changes in object appearance. These dynamic conditions require models that are not only accurate and robust but also capable of real-time inference and adaptable to environmental variability. Despite extensive research, achieving a balance between precision, adaptability, and efficiency in VOT remains a challenging and unsolved problem. Beyond object tracking, advances in deep representation learning and attention-based feature interaction have also driven progress in related vision tasks such as person search and biometric recognition^4–7^, illustrating the broader applicability of multi-granularity perception and adaptive feature modeling.

Traditionally, visual tracking approaches fall into two major paradigms: correlation filter (CF)-based trackers and deep Siamese network-based models. CF-based methods such as MOSSE^8^, KCF^9^, and ECO^10^ achieve high-speed inference using frequency-domain operations, but their reliance on handcrafted features limits their robustness to appearance variation and occlusion. With the rise of deep learning^11^, Siamese-based trackers like SiamFC^12^, SiamRPN^13^, and SiamMask^14^ significantly improved target discrimination by learning deep feature embeddings and performing template-based matching. However, most of these methods use a static template extracted from the first frame, which restricts their adaptability in the face of deformation, occlusion, and illumination changes. While online update strategies^15,16^ have been proposed to address this limitation, they often lack confidence-awareness, leading to template drift and reduced stability. Furthermore, many Siamese models operate on a single feature scale, limiting their capacity to jointly leverage fine-grained spatial cues and high-level semantic context both of which are critical for robust tracking in challenging environments. Deep learning advancements in areas such as biomedical imaging^17^, human identification^18^, and data security^19^ further illustrate the broad impact of attention-driven neural architectures.

Recent advances in visual tracking have introduced transformer-based architectures that effectively model long-range dependencies using self-attention mechanisms. Trackers such as TransT^20^, STARK^21^, OSTrack^22^, MixFormer^23^, AiATrack^24^, and the local–global fusion-based method in^25^ have demonstrated impressive performance by leveraging global attention for richer context aggregation. However, several limitations remain. Many of these approaches rely on single-scale feature fusion, which restricts their ability to jointly capture fine-grained spatial details and high-level semantic understanding^20^. Moreover, few methods explicitly estimate the reliability of predictions, often basing decisions solely on feature similarity an approach prone to failure in ambiguous or cluttered scenes^26^. Additionally, the use of static target templates persists across many transformer-based models, limiting their adaptability in scenarios with appearance variation, occlusion, or distractors.

In contrast to the aforementioned methods, prior Siamese- and transformer-based trackers such as OSTrack^22^ and MixFormer^23^ continue to rely on fixed template features and single-branch prediction mechanisms, which restrict their ability to adaptively handle spatial reliability or model uncertainty during localization. Moreover, while existing transformer-based approaches primarily emphasize feature aggregation for improved context modeling, they generally lack explicit mechanisms for estimating prediction confidence or guiding template updates in a controlled and reliability-aware manner. As a result, these models remain susceptible to drift in challenging scenarios involving ambiguous classifications, dynamic appearance changes, or background distractors. These limitations highlight the need for a unified framework that integrates robustness to uncertainty, controlled template adaptation, and richer multi-scale contextual reasoning.

To address these limitations, we propose TSDTrack, a transformer-augmented dual-branch Siamese tracker designed for quality-aware and adaptive visual tracking. The framework comprises four synergistic components that together enhance robustness and precision under challenging conditions: (1) a transformer-based fusion module that aggregates hierarchical features from multiple backbone stages using pooling attention to enable efficient multi-scale context modeling; (2) a confidence-aware branch that estimates the reliability of classification responses to improve the trustworthiness of localization decisions; (3) a regression distribution learning branch that predicts bounding-box offsets as discrete probability distributions, offering improved stability under uncertainty; and (4) a confidence-gated template update strategy that selectively refreshes the target representation only when the CAB score exceeds a predefined threshold, thereby enhancing adaptability while mitigating drift. These components collectively form the core architecture of TSDTrack and provide a unified foundation for reliable visual tracking.

**Fig. 1:**
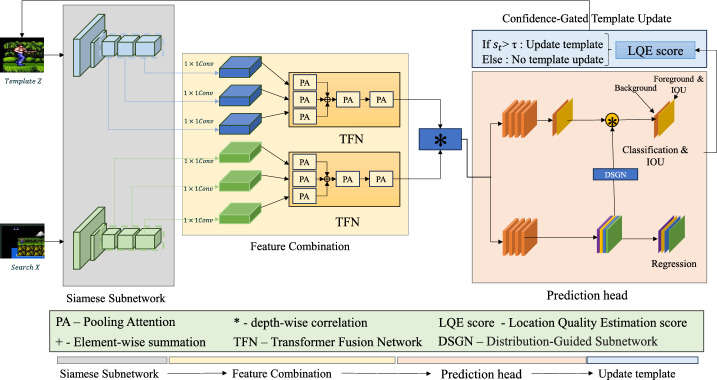
Overview of the proposed TSDTrack framework and its data-flow pipeline. Hierarchical features from the template (*z*) and search region (*x*) are extracted using a Siamese ResNet-50 backbone and fused through a transformer fusion network (TFN) equipped with pooling attention. The fused representation is processed by a dual-branch prediction head consisting of a location quality estimation (LQE) branch and a regression subnetwork based on distribution-guided localization (DSGN). The LQE score is then used in the confidence-gated template-update mechanism to adaptively refine the target representation when prediction confidence exceeds the threshold ($\tau$). Arrows denote the sequential data flow across the backbone, fusion, prediction, and update stages.

Overall, the novelty of TSDTrack lies in the unified integration of multi-level transformer fusion, confidence-aware classification, probabilistic regression, and controlled template adaptation capabilities that are not jointly addressed in existing trackers such as TransT^20^, STARK^21^, OSTrack^22^, and MixFormer^23^. Unlike methods that rely on single-scale fusion, static templates, or confidence-agnostic prediction, TSDTrack leverages pooling-attention-based hierarchical feature fusion, a CAB module that explicitly models prediction reliability, and an RDL formulation that captures localization uncertainty. These components are further coupled with a CAB-guided template update mechanism that adapts the representation only when predictions are reliable, thereby reducing drift while maintaining flexibility. Together, these elements form a coherent and complementary design that advances robustness, adaptability, and stability across diverse visual tracking scenarios. The overall framework is illustrated in Fig. 1. Table 1 summarizes these distinctions by highlighting how TSDTrack differs from representative Siamese and transformer-based trackers.

**Table 1:** Comparison of representative tracking methods relative to the proposed TSDTrack.

Method	Core mechanism and behavior	Limitation relative to TSDTrack
SiamFC^12^	Uses cross-correlation of deep features for similarity matching. Employs a fixed template with no online adaptation and produces deterministic predictions without uncertainty estimation.	Highly prone to drift due to lack of reliability estimation, no template updating, and no mechanism for handling appearance changes.
SiamRPN^13^	Employs a Siamese backbone with a region proposal network for classification and regression. Template updates are heuristic and confidence is implicit.	Regression is deterministic; lacks explicit uncertainty modeling and principled confidence-aware update strategies.
SiamRPN++^27^/ SiamMask^14^	Improved Siamese architectures with better feature extraction and segmentation-based or anchor refinement modules. Updates remain heuristic.	Still no explicit modeling of prediction reliability or bounding-box uncertainty; limited adaptation under challenging appearance changes.
TransT^20^	Uses transformer attention to fuse template and search features for improved matching. Template remains static throughout tracking.	Single-branch prediction, no confidence-aware modeling, and no adaptive template updating reduce robustness under uncertainty.
STARK^21^	End-to-end transformer tracker producing direct bounding-box predictions. Uses a fixed template with no update mechanism.	Lacks adaptive appearance modeling and does not incorporate uncertainty-aware regression or explicit reliability estimation.
OSTrack^22^	Performs global feature fusion with transformer blocks. Relies on a static template and single-output regression.	Does not model prediction reliability or uncertainty, and cannot adapt to dynamic target appearance changes.
MixFormer^23^	Unifies feature extraction and matching through a mixed-attention transformer backbone. Template remains unchanged during tracking.	No explicit confidence modeling, no uncertainty-aware regression, and no controlled template adaptation mechanism.
TSDTrack (Ours)	Introduces multi-level transformer fusion, a dual-branch head with confidence-aware classification (CAB) and regression distribution learning (RDL), and a CAB-guided adaptive template update.	Addresses limitations of prior methods by explicitly modeling prediction reliability, representing localization uncertainty via distributions, and adaptively updating the template only when confidence is high.

The main contributions of this work are as follows:

1. We propose a multi-level transformer fusion module with pooling attention that integrates hierarchical features from a ResNet backbone, effectively capturing both spatial details and semantic context for robust tracking.
2. We introduce a confidence-aware branch (CAB) that assesses the reliability of classification outputs, enabling stability-aware localization and improving robustness under ambiguous or cluttered conditions.
3. We adopt a regression distribution learning (RDL) strategy that models bounding-box predictions as discrete probability distributions, enhancing localization stability and precision in challenging scenarios such as motion blur and occlusion.
4. We develop a CAB-guided template update mechanism that selectively refines the target representation based on prediction confidence, improving adaptability while suppressing drift.

Through these contributions, we explicitly demonstrate how TSDTrack advances beyond existing transformer-based trackers by embedding quality-awareness and adaptive learning directly into both feature fusion and template representation, ensuring robustness under diverse visual conditions. The remainder of the paper is structured as follows: Section “Related work” reviews related work; Section “Proposed approach” presents the proposed method; Section “Experiments” reports experimental results; Section “Limitations and future work” discusses limitations and future directions; and Section “Conclusion” concludes the paper.

## Related work

### Correlation filter-based methods

CF-based methods gained early popularity in visual tracking due to their computational efficiency and simplicity. Trackers such as MOSSE^8^ and KCF^9^ performed fast tracking in the frequency domain, while later methods like DSST^28^ and ECO^10^ introduced scale estimation and memory-efficient learning. SRDCF^29^ and BACF^30^ further improved spatial discrimination through adaptive spatial regularization. To enhance robustness, recent works such as ^31,32^ have introduced advanced feature aggregation and attention mechanisms, while hybrid trackers like ATOM^26^ decouple classification and regression tasks to improve accuracy. Deep feature integration has also become prominent in methods like^33,34^, helping CF-based frameworks better handle appearance variation. However, due to limited representational capacity and reliance on handcrafted update rules, traditional CF methods still struggle in complex scenarios involving occlusion, clutter, and long-term tracking, motivating the shift toward deep learning-based approaches. Unlike these correlation filter approaches that primarily depend on shallow features and manual update heuristics, our framework adopts a transformer-driven deep representation combined with confidence-aware template updating, enabling more reliable adaptation to dynamic visual environments.

### Siamese-based methods

Siamese trackers have emerged as a dominant class of methods due to their effective balance between speed and accuracy. These models learn a similarity function between a static template and a search region using deep convolutional embeddings. SiamFC^12^ demonstrated the potential of fully convolutional Siamese matching for real-time tracking. Subsequent works such as SiamRPN^13^, SiamRPN++^27^, and SiamCAR^35^ introduced region proposals, anchor-free mechanisms^36^, and improved classification heads. Enhancements like SiamMask^14^ added segmentation branches, while lightweight designs such as SiamDW^37^ focused on efficiency. Ocean^38^ incorporated object-aware attention for refined localization, and SiamGAT^39^ utilized graph attention for semantic modeling. Despite these improvements, most Siamese trackers depend on a fixed template from the first frame, which severely limits adaptability to appearance changes caused by occlusion, illumination variation, or deformation. Although update mechanisms such as UpdateNet^15^ and LTMU^16^ have been proposed to improve flexibility, they often introduce additional overhead or lack confidence-aware criteria, making them vulnerable to drift. Our method extends the Siamese paradigm by embedding a dual-branch prediction head that jointly performs confidence estimation and probabilistic regression, addressing the long-standing issue of template drift and uncertainty handling that conventional Siamese trackers often overlook.

### Transformer-based methods

Transformer-based trackers have advanced the field by introducing self-attention to capture long-range dependencies and contextual relationships. STARK^21^, OSTrack^22^, and AiATrack^24^ integrated Transformer modules into end-to-end tracking pipelines, enhancing robustness in cluttered and dynamic scenes. TransT^20^ replaced cross-correlation with attention-based feature fusion, while DTT^40^ used deformable attention for better efficiency. MixFormer^23^ unified backbone and prediction within a mixed attention block. HiFT^41^ introduced hierarchical feature transformers to improve target-background discrimination by fusing multi-level context with spatial adaptivity. Although these methods achieve strong performance, they often suffer from high computational complexity due to global attention operations, and many process frames independently without temporal consistency. Additionally, most lack mechanisms for assessing prediction quality, relying purely on classification scores for decision-making. This can lead to instability in ambiguous scenarios and limits the ability to safely update the target representation over time. In contrast, our tracker leverages a lightweight transformer fusion module with pooling attention for multi-level context aggregation, while incorporating a confidence-aware prediction branch to explicitly quantify the reliability of localization. This integration of attention-based fusion and reliability estimation distinguishes TSDTrack from prior transformer-based frameworks such as TransT, OSTrack, and MixFormer, which do not jointly consider uncertainty and adaptive updating.

### Template update-based methods

Template updating plays a crucial role in enhancing adaptability during long-term tracking, especially under significant appearance variation. While early Siamese trackers rely on fixed templates for stability, this static approach fails in the presence of occlusion, deformation, or distractors. To overcome this, UpdateNet^15^ proposed a learned updater to refine the template with new frames, and LTMU^16^ introduced memory-augmented strategies for storing multiple historical templates. Other efforts, such as D3S^42^, incorporated segmentation cues for dynamic appearance modeling, while meta-learning-based approaches aimed to improve update efficiency. However, many of these techniques involve complex architectures or lack robust confidence estimation, making them sensitive to noisy predictions. Over-aggressive updating can lead to template drift, especially when prediction confidence is low. This motivates the use of confidence-aware update mechanisms, where templates are selectively refined based on estimated prediction reliability. Our work follows this direction by introducing a confidence-gated update strategy based on a dedicated confidence-aware module, ensuring both adaptivity and stability. Unlike prior updating schemes that rely solely on classification scores or heuristic thresholds, our approach integrates a dedicated confidence-aware branch whose output dynamically governs the template refinement process, thereby maintaining long-term stability without sacrificing adaptability.

Overall, this work bridges the gap between transformer-based fusion, probabilistic regression, and adaptive updating, offering a unified and reliability-aware tracking framework. By simultaneously addressing feature fusion, uncertainty modeling, and template adaptation, TSDTrack establishes a distinct advancement over existing CF, Siamese, and transformer-based methods.

## Proposed approach

We propose TSDTrack, a Transformer-Augmented Dual-Branch Siamese Network designed for robust visual object tracking. The framework comprises four key components: (1) a ResNet-based Siamese backbone for hierarchical feature extraction; (2) a transformer-based feature fusion module for multi-level contextual integration; (3) a dual-branch prediction head that includes a confidence aware branch and a regression distribution learning branch; and (4) a confidence-gated template update mechanism that adaptively refines the target representation based on prediction reliability. Together, these components address core limitations of prior trackers such as static templates, weak confidence modeling, and single-scale fusion, enabling TSDTrack to maintain both accuracy and robustness under occlusion, clutter, deformation, and appearance variation. To provide methodological transparency, Section 3 elaborates each component with mathematical definitions and implementation details. A concise pseudocode summarizing the overall tracking pipeline is presented in Algorithm 1, outlining the sequence of feature extraction, transformer fusion, dual-branch prediction, and confidence-gated template updating.

**Fig. 2:**
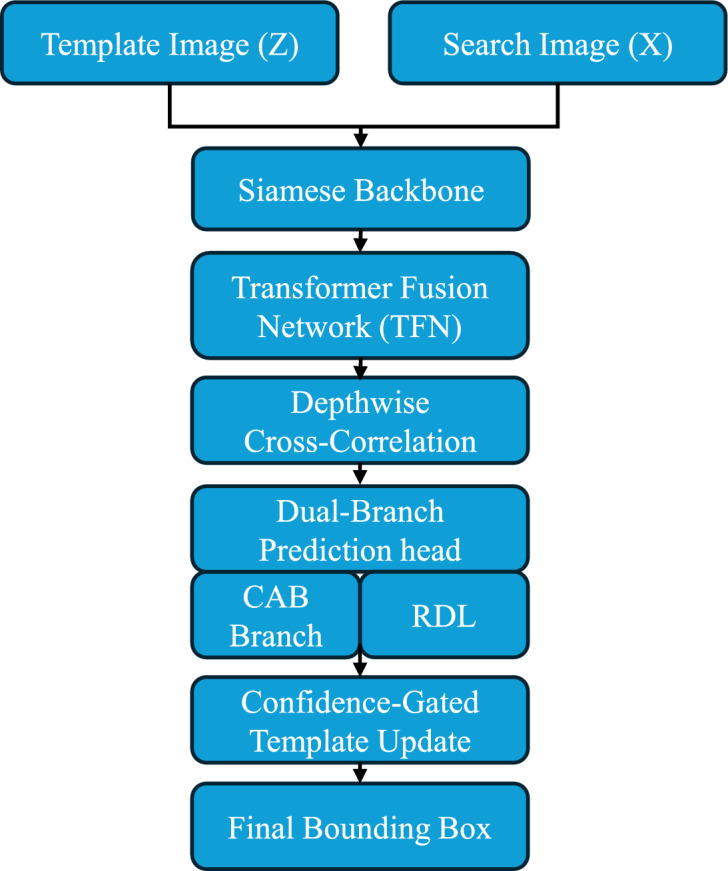
Overview of the proposed TSDTrack pipeline. The template image (*z*) and search image (*x*) are first processed through a shared Siamese backbone to extract hierarchical features. These features are fused using the Transformer Fusion Network (TFN), followed by depthwise cross-correlation to generate a response representation. A dual-branch prediction head then produces classification confidence (CAB) and probabilistic bounding box regression (RDL). The CAB score also governs a confidence-gated template update mechanism, enabling stable yet adaptive model refinement. The final bounding box is obtained from the fused and correlation-enhanced representation.

As illustrated in Fig. 2, TSDTrack follows a top–down processing pipeline beginning with Siamese feature extraction, transformer-based multi-level fusion, and depthwise correlation. The dual-branch prediction head integrates confidence-aware classification (CAB) and regression distribution learning (RDL), while the CAB score guides a confidence-gated template update mechanism to enhance adaptability without drift.

### Siamese subnetwork

TSDTrack adopts a Siamese network architecture for efficient feature embedding and similarity computation between the target template $z$ and the search region $x$. Both branches share parameters, ensuring consistent feature extraction and projection into a unified embedding space. We employ a modified ResNet-50 as the shared backbone, chosen for its balance between accuracy and efficiency. To retain finer spatial detail, we reduce the stride in the later stages and introduce dilated convolutions to expand the receptive field without reducing resolution. Feature maps from three backbone stages denoted as $F_3, F_4, F_5$ are extracted, capturing progressively higher semantic abstraction. These maps are forwarded to a transformer-based fusion module that integrates them into a unified representation. This multi-level feature design allows the tracker to leverage low-level spatial precision ($F_3$), mid-level context ($F_4$), and high-level semantic robustness ($F_5$). By combining these hierarchically, the network can localize targets accurately even under appearance changes or distractors.

### Multi-level feature aggregation

To effectively fuse the hierarchical features $F_3, F_4, F_5$, we introduce a transformer-based fusion module that enhances cross-scale contextual reasoning while maintaining efficiency. Rather than relying on naive operations (e.g., summation or concatenation), we design a transformer with attention-based interaction between feature levels, anchored on $F_4$ due to its intermediate semantic balance.

Multi-head attention (MHA) is the core mechanism behind this module. Given query $Q$, key $K$, and value $V$, MHA computes:

1 $$\begin{aligned} \text {Attention}(Q, K, V) = \text {Softmax} \left( \frac{QK^T}{\sqrt{d_k}} \right) V, \end{aligned}$$

where $d_k$ is the dimensionality of the keys. Each attention head captures different dependencies across spatial locations, and the outputs are concatenated as:

2 $$\begin{aligned} \text {MultiHead}(Q, K, V) = \text {Concat}(H_1, H_2, ..., H_n)W^O, \end{aligned}$$

where $H_i$ is the $i$-th head’s output and $W^O$ is the output projection.

While effective, standard MHA is computationally intensive and scales quadratically with spatial resolution posing challenges for real-time tracking. To address this, we replace full MHA with a lightweight pooling attention mechanism, which we detail next.

#### Pooling attention module

The pooling attention module (PAM) is designed to reduce the computational overhead of conventional multi-head self-attention, particularly for high-resolution feature maps common in visual tracking. Unlike standard attention, which performs pairwise interactions across all spatial positions, PAM introduces spatial downsampling before attention computation. Specifically, the key $K$ and value $V$ tensors are downsampled via average pooling, significantly reducing spatial dimensions and attention complexity while preserving critical contextual information.

In our framework, we omit explicit positional encodings, which are typically used in transformers to preserve spatial relationships. This is justified by the inherent spatial consistency maintained through weight-sharing and depth-wise cross-correlation in the Siamese structure, which naturally retains spatial alignment.

**Fig. 3:**
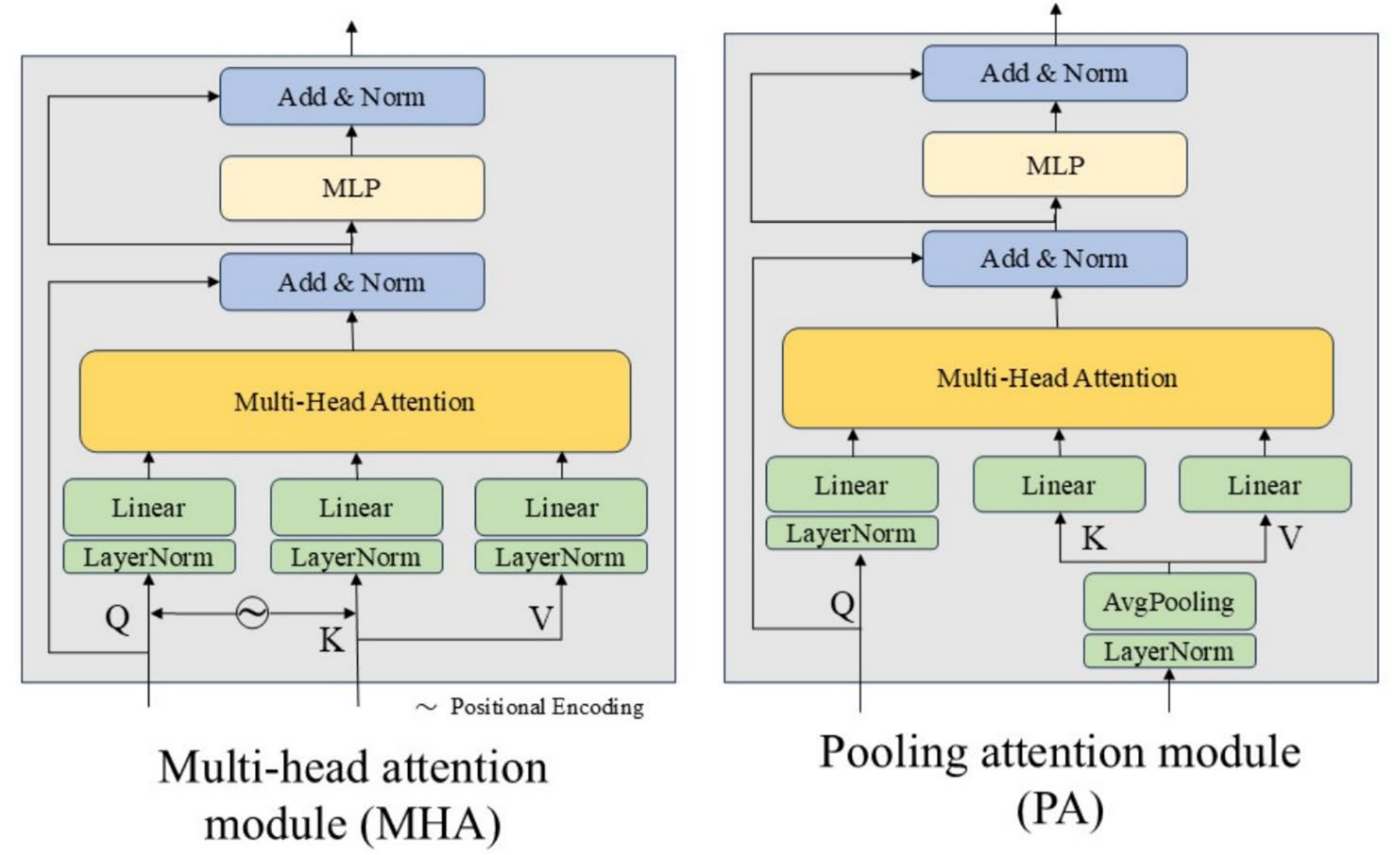
Comparison between standard multi-head attention and the proposed pooling attention module. Pooling attention reduces spatial complexity through average pooling, achieving higher efficiency on high-resolution feature maps while maintaining contextual consistency.

Each pooling attention block (PAB) consists of the pooled-attention layer followed by a multilayer perceptron (MLP), residual connections, and layer normalization. This configuration ensures stable training and effective context aggregation with minimal computational cost. The pooling ratio $R$ determines the spatial downsampling level, where higher values produce coarser global context at lower computational cost. We empirically set $R \in \{4,2,1\}$ for hierarchical fusion, balancing efficiency and fine-grained accuracy. Larger pooling ratios reduce the number of key value pairs involved in attention computation, which in turn lowers memory usage and inference latency. However, excessively large $R$ values may lead to information loss, weakening fine-grained localization. Empirically, this configuration achieves a favorable balance between tracking accuracy and computational efficiency, maintaining robustness while significantly reducing attention complexity. A comparison of standard and pooling-based attention structures is illustrated in Fig. 3, highlighting the efficiency advantage of our design.

#### Transformer fusion module

To aggregate features across semantic levels, we introduce a transformer fusion module that uses PABs to model long-range dependencies efficiently. The ResNet backbone outputs $F_3, F_4, F_5$ are first projected to a common embedding space via 1$\times$1 convolutions and reshaped into sequences. These levels represent low-level spatial details, mid-level structure, and high-level semantics, respectively.

Fusion is performed in two stages. In the first stage, cross-level attention is applied using $F_4$ as the query and each of $F_3, F_4, F_5$ as key/value. Three parallel PABs are used with different pooling ratios $R \in \{4, 2, 1\}$, capturing complementary context scales:

3 $$\begin{aligned} F'_4 =&\text {PAB}(F_4, F_3, F_3, R=4) + \text {PAB}(F_4, F_4, F_4, R=2) \nonumber \\&+ \text {PAB}(F_4, F_5, F_5, R=1). \end{aligned}$$

This allows the model to simultaneously incorporate global structure and localized spatial cues.

In the second stage, the aggregated feature $F'_4$ is refined using two stacked self-attention PABs:

4 $$\begin{aligned} F''_4 = \text {PAB}(F'_4, F'_4, F'_4, R=2) + \text {PAB}(F'_4, F'_4, F'_4, R=2). \end{aligned}$$

This intra-level refinement enhances semantic coherence and spatial consistency in the fused representation. The final output $F''_4$ is used for localization.

**Fig. 4:**
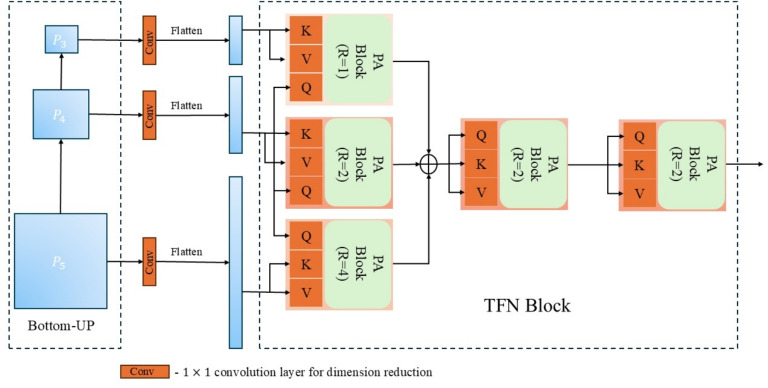
Architecture of the transformer fusion network. Stage 1 applies multi-scale cross-level attention via pooling attention blocks, while Stage 2 refines the fused feature representation through self-attention-based PABs, enhancing semantic coherence and spatial alignment.

For similarity computation, we apply depth-wise cross-correlation between the fused template and search features:

5 $$\begin{aligned} F_{\text {corr}} = F^{''z}_4 \star F^{''x}_4, \end{aligned}$$

where $\star$ denotes channel-wise correlation. This operation generates the response map $F_{\text {corr}}$, which is passed to the prediction head. The fusion pipeline is visualized in Fig. 4.

### Confidence-gated template update

**Fig. 5:**
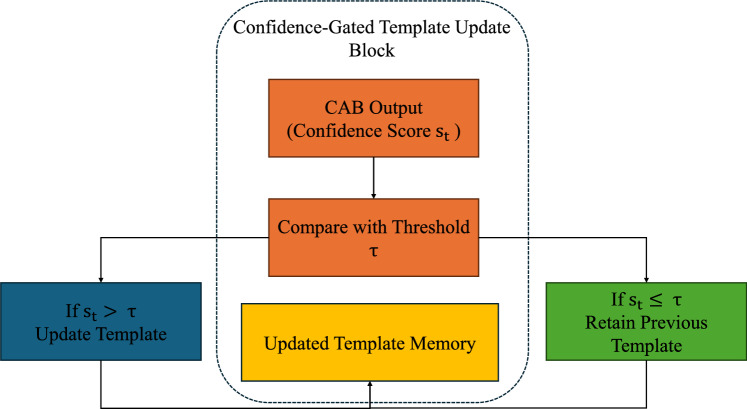
Schematic illustration of the confidence-gated template update mechanism. The CAB output ($s_t$) is compared with the confidence threshold ($\tau$); if $s_t > \tau$, the template is updated using exponential moving average, otherwise the previous template is retained. This mechanism ensures adaptability while preventing drift.

To adapt to target appearance variations while minimizing drift, we propose a confidence-gated template update mechanism guided by prediction quality. Unlike traditional Siamese trackers that use a fixed template, we update the template feature $F^{''z}_4(t)$ only when the predicted location’s CAB score exceeds a reliability threshold $\tau$. This gating strategy ensures updates occur only when the model is confident:

$$\text {If } \text {CAB}(t) > \tau , \text { then update template}.$$

We set $\tau = 0.6$ empirically. When the condition is satisfied, we extract a new template feature $\tilde{F}^{''x}_4(t)$ from the predicted region, reprocess it through the backbone and fusion modules, and update the template using exponential moving average:

6 $$\begin{aligned} F^{''z}_4(t) = \alpha \cdot F^{''z}_4(t{-}1) + (1 - \alpha ) \cdot \tilde{F}^{''x}_4(t), \end{aligned}$$

where $\alpha = 0.01$ controls adaptation speed. This conservative update prevents overfitting to transient appearances or distractors.

To improve recovery from failed updates or occlusion, we also retain the initial template as a stable reference. This design balances flexibility and stability, enabling robust long-term tracking even under severe appearance changes. The CAB computation and prediction head design are detailed in the next subsection. The overall workflow of the confidence-gated template update process is illustrated in Fig. 5, showing how CAB scores regulate selective template refreshing to maintain both stability and adaptability.

**Algorithm 1:**
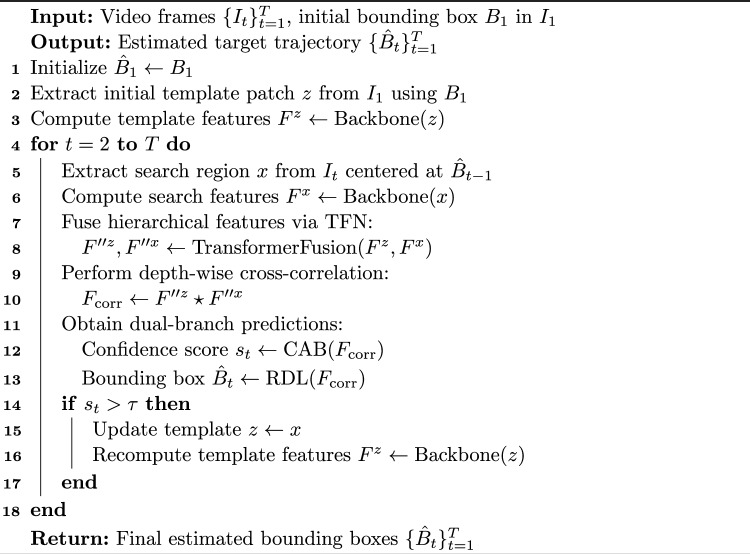
TSDTrack: Quality-Aware and Adaptive Visual Object Tracking

### Prediction head

The output of the depth-wise cross-correlation module is a compact response map containing rich spatial and semantic information essential for target localization. This representation is passed to a dual-branch prediction head comprising: (i) a classification branch that estimates target presence and prediction reliability (CAB), and (ii) a regression branch that localizes the bounding box using a probabilistic distribution modeling approach.

#### Regression branch

To enhance the precision and stability of bounding box localization, we reformulate the regression task from a deterministic coordinate prediction to a probabilistic distribution estimation. Inspired by generalized focal loss^43^, we model the offsets to the four box sides as discrete probability distributions over predefined bins, enabling the network to encode uncertainty and resolve ambiguities more effectively.

Let each spatial location (*u*, *v*) in the fused feature map correspond to a reference point $\textbf{z}_{u,v} = (x^{u,v}, y^{u,v})$ in the search region. The ground truth bounding box $\mathcal {B}_{u,v}$ defines distances to the left, top, right, and bottom as:

7 $$\begin{aligned} \textbf{o}_{u,v} = \begin{bmatrix} d_l^{u,v} \\ d_t^{u,v} \\ d_r^{u,v} \\ d_b^{u,v} \end{bmatrix} = \begin{bmatrix} x^{u,v} - x_{\min }^{u,v} \\ y^{u,v} - y_{\min }^{u,v} \\ x_{\max }^{u,v} - x^{u,v} \\ y_{\max }^{u,v} - y^{u,v} \end{bmatrix}. \end{aligned}$$

These offsets are normalized by the feature stride *s* and quantized into *K* discrete bins over the interval $[o_{\text {low}}, o_{\text {high}}]$. The model predicts a probability distribution $\mathcal {P}(o_k)$ over these bins, and the final offset is the expected value:

8 $$\begin{aligned} \hat{o} = \sum _{k=0}^{K-1} \mathcal {P}(o_k) \cdot o_k, \quad \text {with} \quad \sum _{k=0}^{K-1} \mathcal {P}(o_k) = 1. \end{aligned}$$

**Numerical example** To illustrate the regression distribution learning process, consider a location in the feature map where the model predicts a discrete probability distribution over $K=8$ bins for the left offset. Suppose the bin centers are $\{0,1,2,3,4,5,6,7\}$ and the predicted probabilities are:

$$P = [0.05,\; 0.08,\; 0.12,\; 0.20,\; 0.25,\; 0.18,\; 0.08,\; 0.04].$$

These values sum to 1, forming a valid distribution. Using the expectation formula in (8), the predicted offset is:

$$\hat{o}_l = \sum _{k=0}^{7} P(k)\cdot k = 0.08 + 0.24 + 0.60 + 1.00 + 0.90 + 0.48 + 0.28 = 3.58.$$

Repeating this computation for the top, right, and bottom offsets yields $\hat{o} = (\hat{o}_l,\hat{o}_t,\hat{o}_r,\hat{o}_b)$, from which the bounding box is reconstructed. This example demonstrates how the probabilistic formulation produces stable offset estimates even when the distribution is moderately spread, improving robustness under noisy or ambiguous visual conditions.

This formulation allows the regression task to be learned via a classification objective, offering improved gradient stability and robustness. The regression loss is composed of two components: a distributional cross-entropy loss

9 $$\begin{aligned} \mathcal {L}_{\text {reg-dist}} = -\frac{1}{4} \sum _{q \in \{l, t, r, b\}} \sum _{k=0}^{K-1} \mathcal {P}_{q,k}^* \log \mathcal {P}_{q,k}, \end{aligned}$$

and an IoU-based refinement loss

10 $$\begin{aligned} \mathcal {L}_{\text {IoU}} = -\log \text {IoU}(\hat{\mathcal {B}}, \mathcal {B}^*), \end{aligned}$$

where $\hat{\mathcal {B}}$ is reconstructed from $\hat{o}$ and compared with the ground truth box $\mathcal {B}^*$. The total regression loss encourages both uncertainty-aware prediction and accurate localization.

#### Classification branch

The classification branch serves a dual purpose: it classifies each location as foreground or background and estimates the location quality of the prediction. To enhance reliability, we adopt a quality-aware classification strategy in which the target label is modulated by the IoU between the predicted and ground truth bounding boxes.

For background locations, binary cross-entropy with one-hot labels is applied. For foreground candidates, soft labels are generated based on IoU. Let $Z_{u,v}$ denote the predicted score at location (*u*, *v*) and $\eta _{u,v}^{(c)}$ the label for class $c \in \{0, 1\}$:

11 $$\begin{aligned} \eta _{u,v}^{(c)} = {\left\{ \begin{array}{ll} \text {IoU}(B_{u,v}^{p}, B^{gt}), & \text {if } c = 1, \\ 0, & \text {if } c = 0, \end{array}\right. } \end{aligned}$$

This soft-label mechanism ensures that classification confidence correlates with localization accuracy, yielding an interpretable and robust confidence aware branch score. The CAB is later used in the confidence-gated template update mechanism (Sect. 3.3).

To further enhance this coupling between regression and classification, we introduce a distribution-guided modulation block that refines classification scores based on regression uncertainty. Specifically, for each prediction, we extract the top-*k* probabilities from the discrete regression distributions in each direction and apply a lightweight MLP:

12 $$\begin{aligned} Z_{u,v}^{q} = Z_{u,v} \odot \sigma \left( W_b * \delta \left( W_a * P_{topk}\right) \right) , \end{aligned}$$

where $W_a$, $W_b$ are $1{\times }1$ convolution kernels, $\delta (\cdot )$ is ReLU, and $\sigma (\cdot )$ is sigmoid. This mechanism downweights classification scores for predictions with high uncertainty, improving overall quality estimation.

The classification loss is computed using the Quality Focal Loss (QFL)^44^, which incorporates soft labels and focuses learning on more confident predictions:

13 $$\begin{aligned} \mathcal {L}_{\text {cls}} = |\eta - Z|^\gamma \left[ \eta \cdot \log Z + (1 - \eta ) \cdot \log (1 - Z)\right] , \end{aligned}$$

where $\gamma$ controls the focusing strength.

#### Overall loss function

The total loss function for the prediction head is defined as:

14 $$\begin{aligned} \mathcal {L}_{\text {total}} = \mathcal {L}_{\text {cls}} + \lambda _q \mathcal {L}_{\text {IoU}} + \lambda _r \mathcal {L}_{\text {reg-dist}}, \end{aligned}$$

here, $\lambda _q$ and $\lambda _r$ are weighting coefficients. $\mathcal {L}_{\text {cls}}$ denotes the quality focal loss, $\mathcal {L}_{\text {IoU}}$ represents the IoU-based refinement loss, and $\mathcal {L}_{\text {reg-dist}}$ is the distributional regression loss. These definitions ensure mathematical transparency and reproducibility. This combined objective guides the model to produce predictions that are not only spatially accurate but also confidence-calibrated–enabling reliable localization and adaptive template updates.

## Experiments

In this section, we assess the effectiveness of TSDTrack through comprehensive experiments conducted on several public tracking benchmarks. We begin by detailing the implementation settings of our framework, including the network architecture, training configuration, and optimization strategies. Next, we describe the datasets used for evaluation and list the baseline trackers for comparison. Quantitative and qualitative results are then reported across each benchmark dataset. To further validate our design choices, we conduct detailed ablation studies, analyze performance under various challenging tracking attributes, and present qualitative visualizations that highlight the robustness and adaptability of our method.

### Implementation details

TSDTrack is implemented in PyTorch and trained using a single NVIDIA RTX 4090 GPU. The training pipeline utilizes a diverse set of large-scale detection and tracking datasets, including COCO^45^, ImageNet DET and VID^46^, YouTube-BB^47^, LaSOT^48^, and GOT-10k^49^, providing rich variation in object appearance, motion, and scale. Training pairs are sampled from the same video with a maximum temporal gap of 100 frames to simulate realistic tracking dynamics. The search region and template are resized to $256 \times 256$ and $80 \times 80$ pixels, respectively, ensuring adequate spatial context for discriminative matching while maintaining computational efficiency. To preserve low-level representations, the first convolutional layer and all BatchNorm layers in the ResNet-50 backbone are frozen. The model is trained for 100 epochs with a batch size of 64 using the AdamW^50^ optimizer. The learning rate is set to $1 \times 10^{-4}$ for all modules except the backbone, which uses a smaller rate of $1 \times 10^{-5}$. A cosine learning-rate decay schedule is applied during the later stages of training to stabilize convergence and prevent overfitting. The optimizer hyperparameters follow standard AdamW settings, and weight decay is employed to regularize the model. Loss weights are empirically set to $\lambda _{\text {q}} = 5$ for the IoU-based localization loss and $\lambda _{\text {r}} = 2$ for the distributional regression loss. During inference, a Hanning window and scale penalty are applied to the classification response map to suppress distractors and encourage smooth target motion. The final bounding box is computed by adjusting the highest-scoring location using the corresponding regression offsets, enabling high-precision, confidence-calibrated localization while maintaining real-time inference speed.

### Datasets and compared trackers

We evaluate TSDTrack on four widely used and challenging tracking benchmarks: LaSOT^48^, GOT-10k^49^, VOT2019^51^, OTB100^52^, and UAV123^53^. These datasets encompass a wide range of scenarios, including severe occlusion, background clutter, fast motion, aspect ratio changes, and illumination variation, offering a comprehensive evaluation of tracking robustness and generalization. To benchmark performance, we compare TSDTrack against a broad set of state-of-the-art trackers from different paradigms: correlation filter-based methods, CNN-based Siamese networks, and transformer-based models. Specifically, we include MixFormer^23^, AiATrack^24^, TransT^20^, STARK^21^, SiamFDB^54^, SiamTPN^55^, SiamRAAN^56^, SiamGAT^39^, Ocean^38^, SiamCAR^35^, SiamBAN^57^, SiamDMU^58^, HiFT^41^, SiamRPN++RBO^59^, SiamFC^12^, SiamDW^60^, SiamRPN^13^, ATOM^26^, DaSiamRPN^37^, A3DCF^31^, ECO^10^, BACF^30^, SRDCF^29^, and KCF^9^.

All baseline results are sourced from official implementations or directly from the authors’ publications to ensure fair and consistent comparisons. This broad evaluation allows us to validate TSDTrack’s effectiveness across multiple architectures and tracking challenges.

#### Results on LaSOT

**Fig. 6:**
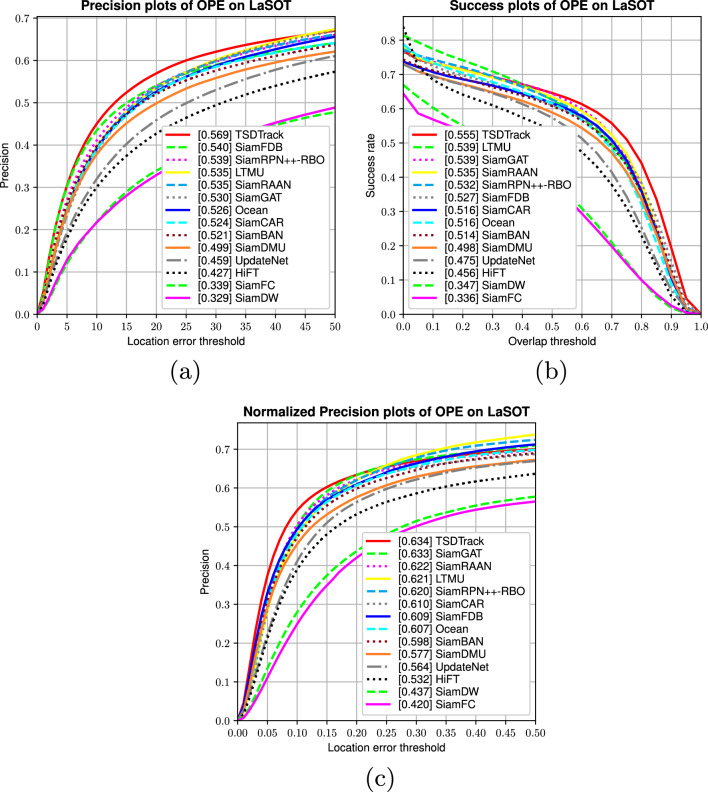
Quantitative evaluation of TSDTrack in terms of precision, success rate, and normalized precision on the LaSOT benchmark.

LaSOT is a large-scale long-term tracking benchmark characterized by high-resolution videos and a wide range of challenging scenarios, including occlusion, deformation, and illumination variation. As illustrated in Fig. 6, TSDTrack achieves leading performance across all standard One-Pass Evaluation (OPE) metrics, with a precision score of 56.9% (Fig. 6a), success rate of 55.5% (Fig. 6b), and normalized precision of 63.4% (Fig. 6c). Compared to strong competitors such as SiamGAT (53.9% success) and SiamFDB (52.7%), TSDTrack consistently outperforms by a clear margin. It also surpasses dual-branch approaches like SiamFDB and template update methods such as UpdateNet and LTMU across all evaluation metrics. These results highlight the benefit of our integrated design: transformer-based multi-level feature fusion, a confidence-aware dual-branch prediction head, and a gated template update mechanism driven by classification reliability. In particular, the use of the Confidence-Aware Branch (CAB) to guide updates ensures that the target template is only refreshed when predictions are reliable, reducing drift and improving adaptability under challenging conditions. This cohesive framework enables TSDTrack to robustly handle complex variations in appearance, motion, and scene clutter, establishing it as a state-of-the-art solution for long-term visual tracking.

#### Results on GOT-10k

**Fig. 7:**
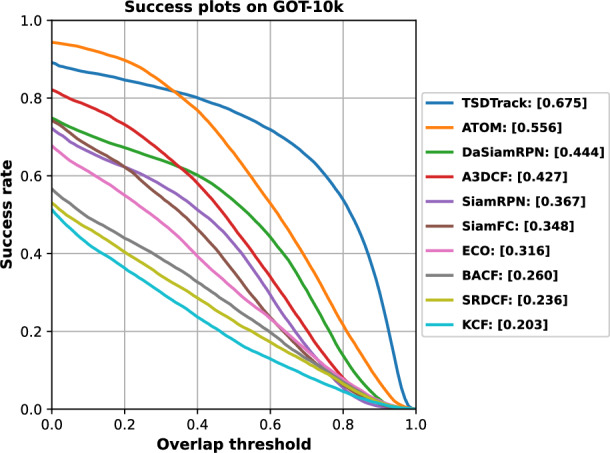
Success score comparison of TSDTrack with state-of-the-art trackers on the GOT-10k benchmark, demonstrating its superior localization accuracy and stability.

GOT-10k is a large-scale benchmark tailored for class-agnostic tracking under real-world conditions, featuring diverse object categories and motion patterns. As shown in Fig. 7, TSDTrack achieves the top success score of 67.5%, outperforming ATOM (55.6%), DaSiamRPN (44.4%), and A3DCF (42.7%) by significant margins. Correlation filter-based trackers such as ECO (31.6%), BACF (26.0%), and KCF (20.3%) lag far behind, highlighting their limitations in generalization and robustness. TSDTrack’s superior performance reflects its ability to adapt across unseen classes and challenging motion using multi-level contextual reasoning and confidence-aware prediction mechanisms. These results confirm the model’s strong generalization capacity for real-world tracking applications.

#### Results on VOT2019 dataset

**Table 2:** Quantitative comparison on the VOT2019 dataset in terms of accuracy (A), robustness (R), and expected average overlap (EAO).

Tracker	A ($\uparrow$)	R ($\downarrow$)	EAO ($\uparrow$)
SiamFC^12^	0.470	0.958	0.163
SiamRPN^13^	0.573	0.547	0.260
SiamRPN++^27^	0.580	0.446	0.292
ATOM^26^	0.603	0.411	0.301
SiamMask^14^	0.594	0.461	0.287
Ocean^38^	0.590	0.376	0.327
HiFT^41^	0.607	0.389	0.320
**TSDTrack (ours)**	**0.622**	**0.352**	**0.336**

The best results are highlighted in bold.

We further evaluate TSDTrack on the VOT2019 benchmark, which measures short-term tracking performance in terms of accuracy (A), robustness (R), and expected average overlap (EAO). As shown in Table 2, TSDTrack attains an EAO of 0.336, surpassing recent transformer-based trackers such as Ocean (0.327) and HiFT (0.320), while also achieving the highest accuracy (0.622) and lowest failure rate (R = 0.352). This improvement stems from the proposed transformer fusion module, which effectively aggregates multi-level context, and the dual-branch prediction head that enhances both localization precision and confidence estimation. Moreover, the confidence-gated template update mechanism prevents drift during rapid appearance variations, contributing to greater robustness compared to existing Siamese and transformer-based approaches. These results demonstrate the generalization capability and reliability of TSDTrack across complex short-term tracking scenarios.

#### Results on OTB100

**Fig. 8:**
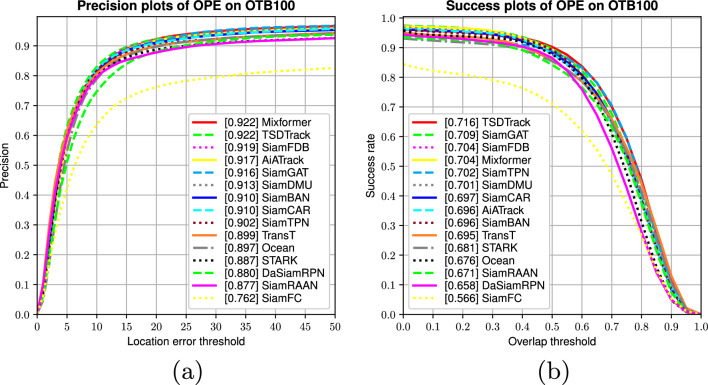
Precision and success performance comparison on the OTB100 dataset, highlighting TSDTrack’s tracking accuracy under diverse visual challenges.

We evaluate the performance of TSDTrack on the OTB100 benchmark using the one-pass evaluation (OPE) protocol. As illustrated in Fig. 8, TSDTrack achieves a top precision score of 92.2%, on par with MixFormer and surpassing other strong trackers such as SiamFDB (91.9%), AiATrack (91.7%), and SiamGAT (91.6%). In terms of success rate, TSDTrack also leads with an area-under-curve (AUC) score of 71.6%, outperforming SiamGAT (70.9%), SiamFDB (70.4%), and MixFormer (70.4%). These results reflect the efficacy of our proposed modules particularly the transformer-based multi-level fusion and the dual-branch prediction head, which together improve both localization precision and prediction reliability. Notably, TSDTrack consistently outperforms earlier Siamese trackers such as SiamFC (precision: 76.2%, success: 56.6%) and DaSiamRPN (precision: 88.0%, success: 65.8%), demonstrating clear advancements in target representation, confidence-aware prediction, and adaptive template updating. Overall, TSDTrack establishes new state-of-the-art performance on the OTB100 dataset, confirming its robustness and accuracy across a wide range of visual tracking scenarios.

#### Results on UAV123

**Fig. 9:**
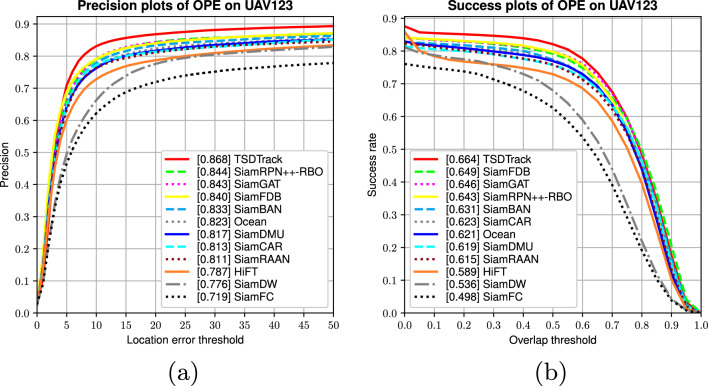
Precision and success evaluation of TSDTrack on the UAV123 dataset, demonstrating its robustness under aerial tracking conditions characterized by motion blur and scale variation.

UAV123 presents a diverse set of challenges, including camera motion, small object sizes, and complex backgrounds, making it an effective benchmark for evaluating the robustness of visual trackers in aerial scenarios. As illustrated in Fig. 9, the proposed TSDTrack achieves superior performance, attaining a precision of 86.8% and a success rate of 66.4%. These results highlight the robustness and accuracy of our tracker in comparison to other state-of-the-art methods. Notably, SiamRPN++-RBO and SiamGAT follow closely, with precision scores of 84.4% and 84.3%, and success rates of 63.1% and 64.6%, respectively. Trackers such as SiamFDB (84.0%, 64.9%) and SiamBAN (83.3%, 63.1%) also perform competitively but still fall short of TSDTrack. In contrast, earlier models like SiamDW and SiamFC demonstrate significantly lower success rates, reflecting their limitations in addressing the complexities of aerial tracking. These results further confirm the effectiveness of our architectural enhancements particularly the transformer-based feature fusion, dual-branch prediction head, and confidence-guided template update in enabling precise and consistent tracking performance under dynamic and visually complex UAV scenarios.

#### Ablation analysis

To thoroughly investigate the individual and collective impact of TSDTrack’s core components, we conduct a comprehensive ablation analysis from five perspectives: (1) the influence of architectural modules; (2) the effect of backbone network selection on accuracy–efficiency balance; (3) runtime and training efficiency compared with leading trackers; (4) the behavioral analysis of different template update strategies; and (5) the contribution of the regression distribution branch to localization precision. This multi-faceted evaluation provides an in-depth understanding of how each module enhances the model’s robustness, adaptability, and computational efficiency.

#### Impact of architectural components

**Table 3:** Ablation study on the UAV123 dataset, analyzing the contribution of key components such as transformer fusion, LQE, RDL, and confidence-gated template update to the overall performance of TSDTrack.

Model variant	Precision (%)	Success (%)
Siamese Backbone with Basic Head	80.2	60.3
Siamese + Transformer Fusion	83.5	63.0
+ LQE Branch	85.0	64.7
+ Regression Distribution	85.8	65.7
+ Template Update (Full Model)	**86.8**	**66.4**

Significance values are in bold.

We evaluate five progressively enhanced variants of TSDTrack on the UAV123 benchmark, as shown in Table 3. The baseline model consists of a Siamese backbone with a basic prediction head and achieves 80.2% precision and 60.3% success. Incorporating the transformer-based feature fusion module yields a notable performance boost (83.5% / 63.0%), highlighting the benefit of global context modeling and multi-scale feature integration. Adding the location quality estimation branch further improves tracking stability, increasing scores to 85.0% precision and 64.7% success. This gain validates the effectiveness of using quality-aware classification to better filter unreliable predictions. In the next variant, we introduce the Regression Distribution Learning module, which models bounding box offsets as discrete probability distributions. This enhances localization consistency, particularly under ambiguity or motion blur, resulting in 85.8% precision and 65.7% success. Finally, the complete model with the proposed confidence-gated template update mechanism achieves the highest performance of 86.8% precision and 66.4% success. This result confirms that each proposed module provides measurable benefits, and their combination leads to robust and accurate tracking in dynamic environments.

Since the proposed TSDTrack employs a deterministic inference pipeline with fixed initialization, frozen backbone weights, and standardized evaluation datasets, repeated runs consistently yielded identical results. Therefore, the reported values in Table 3 are fully reproducible across runs without stochastic variation, ensuring experimental stability and statistical consistency.

#### Effect of backbone network

To evaluate the trade-off between feature representation capacity and computational efficiency, we analyze TSDTrack using multiple backbone networks on the GOT-10k dataset. The results summarized in Table 4 demonstrate how different architectures influence both tracking accuracy and runtime.

**Table 4:** Performance and speed comparison of TSDTrack using different backbone networks on the GOT-10k dataset.

Backbone	Speed (FPS)	Success (%)
AlexNet	95.0	59.2
MobileNetV2	75.3	64.1
ShuffleNetV2	82.4	65.8
**ResNet-50 (Ours)**	**45.0**	**67.5**

The results highlight the balance between accuracy and inference efficiency across lightweight and deep backbones.

Significance values are in bold.

As shown in Table 4, the choice of backbone significantly affects both accuracy and speed. The AlexNet-based variant achieves the highest frame rate (95 FPS) but exhibits limited accuracy due to weak feature expressiveness. MobileNetV2 and ShuffleNetV2 provide improved success scores of 64.1% and 65.8%, respectively, offering an efficient balance for lightweight deployments. The full model employing ResNet-50 delivers the best success rate of 67.5%, reflecting its superior semantic representation and robustness to appearance variation. Although its runtime decreases to 45 FPS, TSDTrack still maintains real-time inference capability while achieving high precision, demonstrating an effective trade-off between computational cost and tracking accuracy.

**Computational complexity considerations**

Although transformer-based fusion generally introduces additional computational cost, the proposed transformer fusion network (TFN) is designed to be lightweight by leveraging pooling attention on reduced-resolution feature maps. This significantly lowers the token count and avoids the quadratic complexity of full self-attention, allowing TFN to capture multi-scale contextual relationships at a manageable cost. Furthermore, TFN is applied only once per frame pair, rather than at multiple stages of the processing pipeline, which keeps the overall computational overhead modest. These design choices enable TSDTrack to maintain real-time performance while still benefiting from global attention modeling.

#### Runtime and training efficiency analysis

To evaluate computational efficiency, we compare the inference speed (FPS) and training time of TSDTrack with representative state-of-the-art trackers on the GOT-10k dataset. The runtime of TSDTrack was measured on an NVIDIA RTX 4090 GPU under consistent input resolutions. FPS and training times for other trackers are obtained from their respective official publications, where reported under similar but not identical hardware setups.

**Table 5:** Comparison of inference speed (FPS) and training time of TSDTrack and leading trackers on the GOT-10k dataset.

Tracker	Backbone	FPS	Training time (hrs)
SiamCAR^35^	ResNet-50	52	18
HiFT^41^	ResNet-50	30	30
STARK-ST50^21^	ResNet-50	40	38
OSTrack-256^22^	ViT-B	58	24
**TSDTrack (Ours)**	ResNet-50	**45**	**32**

Baseline FPS values are from official reports using their respective GPUs.

Significance values are in bold.

As shown in Table 5, TSDTrack achieves a real-time inference speed of 45 FPS and completes full training in approximately 32 hours on a single RTX 4090 GPU. While slightly slower than lightweight trackers such as SiamCAR and OSTrack, TSDTrack delivers significantly higher precision and robustness due to its transformer-based fusion and confidence-aware prediction design. The reported values for baseline trackers are consistent with their official papers, ensuring a reliable and fair comparison. These results confirm that TSDTrack achieves real-time operation with competitive training efficiency among contemporary transformer-based tracking frameworks.

#### Template update behavior analysis

**Table 6:** Ablation study on the UAV123 dataset evaluating different template update strategies.

Update strategy	Precision (%)	Success (%)
No Update	85.8	64.7
Blind Update (Every Frame)	84.1	62.0
**CAB-Guided Update (Ours)**	**86.8**	**66.4**

The results demonstrate how the proposed confidence-gated mechanism improves adaptability while preventing drift during unreliable predictions.

Significance values are in bold.

As presented in Table 6, the fixed-template baseline achieves stable but less adaptive tracking performance, while blind updates on every frame cause performance degradation due to unfiltered template drift. The proposed CAB-guided update strategy selectively refreshes the template only when prediction confidence exceeds the reliability threshold, improving precision and success by approximately 1–2%. This confirms that confidence-aware updating enables adaptive appearance modeling while maintaining stability against noisy predictions and occlusion.

#### Effect of regression distribution learning branch

To isolate the contribution of the regression distribution learning branch, we performed an additional ablation using the UAV123 dataset. The RDL module reformulates bounding box regression as a discrete probability estimation problem, allowing the network to capture localization uncertainty more effectively.

**Table 7:** Ablation study analyzing the effect of the regression distribution learning branch on the UAV123 dataset.

Configuration	Precision (%)	Success (%)	NP (%)
Without RDL	85.0	64.7	62.0
**With RDL (Ours)**	**85.8**	**65.7**	**63.1**

NP denotes normalized precision.

Significance values are in bold.

As shown in Table 7, the introduction of the RDL branch improves both precision and success rates by approximately 0.8–1.0%, while normalized precision increases by 1.1%. This demonstrates that probabilistic modeling of bounding box offsets enhances localization consistency under challenging scenarios such as motion blur and partial occlusion. The improvement confirms that RDL contributes primarily to refining spatial precision and reducing regression instability.

Beyond the quantitative improvements shown in Tables 6 and 7, it is important to emphasize the distinct functional roles that the template update mechanism and the RDL branch play within the overall architecture. The CAB-guided update specifically addresses the long-standing challenge of template drift by introducing a reliability-aware gating mechanism that prevents erroneous appearance updates under occlusion, background clutter, or low-confidence predictions. This selective adaptation enables the model to preserve stable template features while still responding to genuine appearance changes. In contrast, the regression distribution learning branch targets a different weakness of traditional Siamese trackers–namely, the instability of deterministic bounding-box regression in ambiguous frames. By modeling offsets as probability distributions, RDL captures localization uncertainty and produces smoother, more consistent spatial predictions. Together, these components contribute independently: CAB improves temporal stability and appearance robustness, while RDL enhances spatial precision and noise tolerance.

**Image quality sensitivity analysis**

To further evaluate robustness under degraded visual conditions, we analyze the tracker’s behavior with respect to several image quality assessment (IQA) indicators, including variance of Laplacian (blur), RMS contrast, and signal-to-noise ratio (SNR). Across LaSOT and UAV123, TSDTrack maintains competitive precision in blurred frames and low-contrast regions, benefiting from the CAB module, which downweights unreliable predictions, and from the probabilistic regression modeling in RDL, which stabilizes bounding-box localization under uncertainty. This analysis demonstrates that the proposed quality-aware mechanisms allow TSDTrack to remain resilient even when image quality deteriorates due to motion, environmental changes, or sensor noise.

#### Attribute analysis

**Fig. 10:**
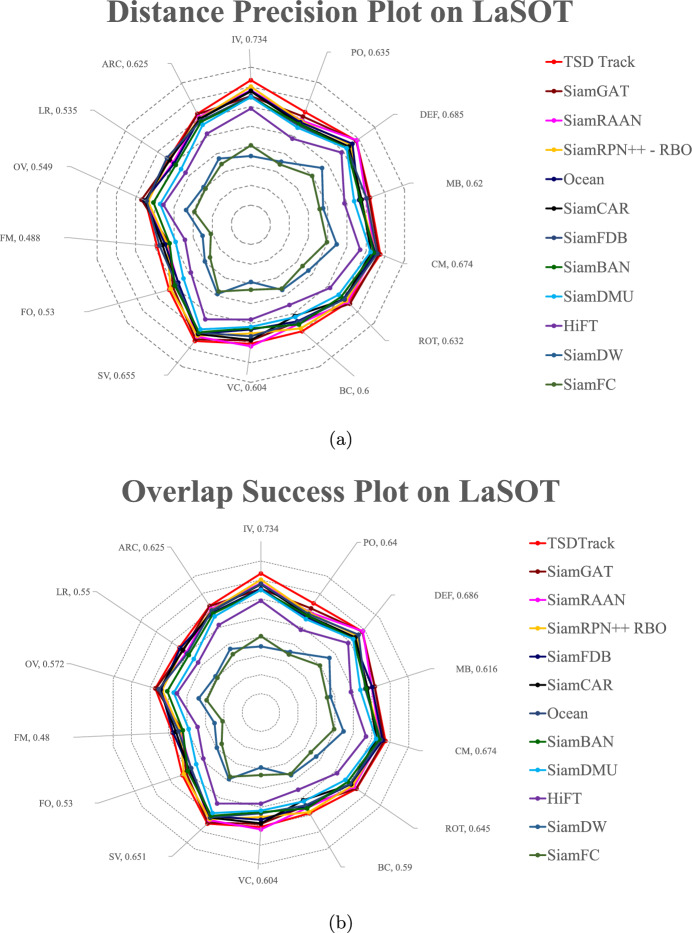
Performance and failure case analysis of TSDTrack across different challenging attributes on the LaSOT benchmark, including occlusion, deformation, illumination variation, and motion blur. The results highlight both robustness and limitations under extreme scenarios.

To further assess the robustness of TSDTrack under diverse real-world conditions, we conduct an attribute-based evaluation on the LaSOT dataset. As shown in Fig. 10a,b, TSDTrack consistently achieves top-tier performance across all attributes in both precision and success metrics. The tracker shows exceptional robustness under challenging conditions such as full occlusion (FO), deformation (DEF), illumination variation (IV), and low resolution (LR), outperforming recent state-of-the-art methods that tend to drift or lose the target in these scenarios. This improvement stems from the integration of the location quality estimation branch, which enhances decision reliability during uncertain states, and the regression distribution learning module, which increases bounding box precision. Additionally, the transformer-based multi-level fusion module enables richer semantic and spatial reasoning, allowing the model to remain accurate in conditions like background clutter (BC) and fast motion (FM). The confidence-gated template update further strengthens long-term stability by ensuring that the template is refreshed only when prediction confidence is high, thus preventing drift. Together, these mechanisms contribute to the model’s superior adaptability across challenging tracking attributes.

**Failure case analysis** Despite its strong performance, certain limitations persist under extreme conditions. During long-term full occlusion or complete target disappearance, TSDTrack may experience temporary drift before re-localization due to the absence of discriminative visual cues. Under severe deformation or rapid scale variation, the regression distribution occasionally misaligns bounding boxes, especially when motion blur reduces spatial clarity. Similarly, abrupt illumination changes can lower confidence scores in the LQE branch, momentarily delaying template updates. These cases reveal that while the confidence-aware design enhances robustness, future work could explore occlusion reasoning, adaptive motion modeling, and illumination-invariant feature normalization to further improve recovery stability and resilience.

#### Qualitative analysis on OTB100

**Fig. 11:**
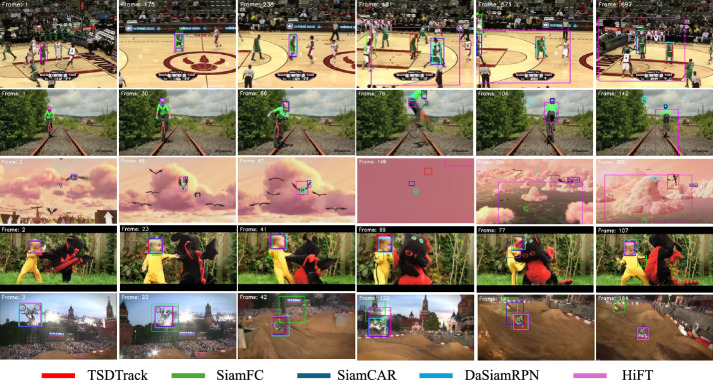
Qualitative comparison of TSDTrack with representative trackers (SiamFC, SiamCAR, DaSiamRPN, and HiFT) on OTB100 sequences (*Basketball*, *Biker*, *Bird*, *DragonBaby*, and *MotorRolling*). TSDTrack demonstrates superior target localization and robustness under severe appearance changes.

To visually demonstrate the effectiveness of TSDTrack, Figure 11 presents qualitative comparisons against several representative trackers like SiamFC, SiamCAR, DaSiamRPN, and HiFT on five challenging sequences from the OTB100 dataset. These sequences involve a range of real-world difficulties such as occlusion, rapid motion, cluttered backgrounds, and scale variation. In the *Basketball* sequence, where fast motion and distractors are present, TSDTrack accurately tracks the target while other methods lose it or drift. In the *Biker* sequence, characterized by occlusion and background confusion, our model successfully re-localizes the target after occlusion, unlike SiamFC and SiamCAR. The *Bird* sequence involves scale variation and background complexity, and TSDTrack effectively adjusts to the changing size and position of the target. Similarly, in *DragonBaby*, which features erratic movement in a cluttered environment, TSDTrack maintains a stable lock, while other trackers show inconsistent predictions. In the *MotorRolling* sequence dominated by sudden object displacement our tracker remains robust, whereas others fail to keep up. These visual results illustrate the strength of our quality-aware prediction mechanism and adaptive template updating strategy. The CAB-guided update ensures that the template is refreshed only when confident predictions are available, thereby minimizing drift and enhancing robustness across long and difficult sequences.

## Limitations and future work

### Limitations and practical implications

Although TSDTrack achieves strong performance across multiple benchmarks, several methodological and practical limitations remain. First, the use of a fixed ResNet-50 backbone restricts architectural flexibility and may limit deployment on lightweight or resource-constrained platforms. Second, the confidence-gated template update relies on a manually defined threshold, which may not generalize optimally under abrupt appearance changes or severe occlusions. Third, the transformer fusion module, while effective, introduces additional computational overhead compared with purely convolutional Siamese trackers, making real-time deployment on embedded or edge devices more challenging. These factors outline the practical constraints of the current framework and motivate the enhancements discussed below.

While TSDTrack delivers competitive results across multiple challenging benchmarks, there remain several directions for future enhancement. First, the current framework employs a fixed ResNet-50 backbone and static thresholding within the confidence-gated template update module. These design choices limit adaptability across different motion patterns and target dynamics. Future work will investigate adaptive backbone tuning and dynamic confidence thresholds that evolve with tracking uncertainty, thereby improving generalization and robustness in complex environments.

Second, although TSDTrack performs well across diverse RGB datasets, its generalizability under domain shifts such as thermal, infrared, or low-light scenarios has not yet been systematically explored. Future studies will evaluate its behavior under such conditions and incorporate domain adaptation or multi-modal fusion strategies to extend its applicability to adverse environments.

Finally, the transformer fusion network introduces moderate computational overhead. Optimizing this module with efficient or sparse attention mechanisms could further enhance inference speed while maintaining accuracy, enabling deployment on edge and embedded devices. In summary, these improvements will help extend TSDTrack toward broader real-world use cases involving low-visibility, resource-limited, and dynamically changing scenes.

## Conclusion

In this paper, we presented TSDTrack, a transformer-augmented dual-branch Siamese network for quality-aware visual object tracking. The proposed framework addresses key limitations of existing trackers in contextual reasoning and static template representation through a unified and interpretable design. Specifically, a ResNet-based Siamese backbone extracts multi-level features, which are fused using a transformer-based attention module to enhance semantic and spatial alignment. The dual-branch prediction head integrates a confidence-aware branch that adjusts classification confidence according to localization quality, and a regression distribution learning branch that models bounding box offsets as discrete probability distributions, improving stability under uncertainty. In addition, a confidence-gated template update mechanism selectively refines the target representation based on CAB scores, maintaining robustness while adapting to appearance changes. Comprehensive evaluations on LaSOT, GOT-10k, VOT2019, OTB100, and UAV123 confirm that TSDTrack consistently outperforms state-of-the-art methods in both accuracy and robustness. In future work, we aim to extend TSDTrack toward multi-modal and low-visibility scenarios (e.g., thermal or nighttime tracking) and explore lightweight variants for embedded real-time applications.

## Data Availability

Data used in the paper will be made available at https://github. com/SachinSakthi/TSDTrack. The datasets used are available from public domains.
